# New products

**DOI:** 10.1155/S1463924603000178

**Published:** 2003


Journal of Automated Methods & Management in Chemistry
Vol. 25, No. 4 (July–August 2003) pp. 101–102

Taylor & Francis

Taylor & Francis Group

### New products

### Chromacol vials

Every day for the last 20 years Chromacol has sold thousands of routine crimp top, screw top and snap cap vials, clear and amber, to both end users and autosampler manufacturers. Why have these people continued to buy these vials from Chromacol? Because they know that to get the best vials they should go to the best supplier.

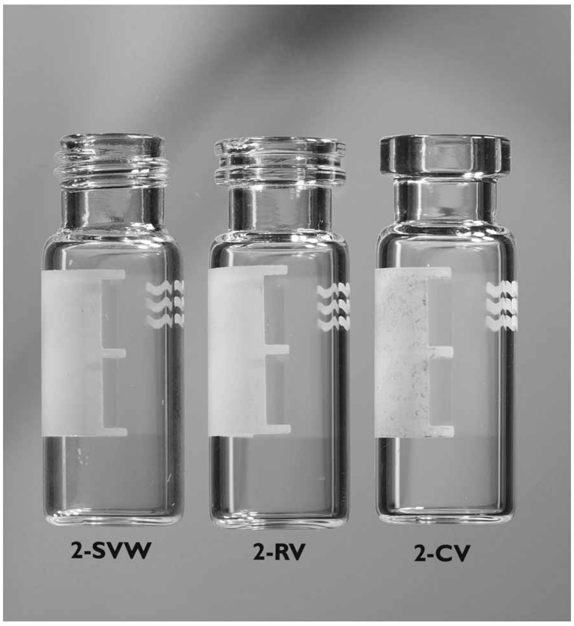

*Routine vials also available in amber*

Chromacol's routine vials can be bought on their own, in combination packs (caps included), or in instrument specific packs called 'Instrument Select' where Chromacol has combined the most suitable vials with the ideal seal and cap combination for a number of popular autosamplers at very competitive prices.
All Chromacol routine vials are supported by Chromacol's 'no quibble' guarantee where the main objective is to ensure continued laboratory productivity with a fast response customer support team.
*For further information contact Chromacol on 0845 7023964 or visit www.chromacol.com*

### Environmentally clean Chromacol vials

Chromacol EPA vials are designed to provide a means of transporting a water sample in the cleanest of environments to the laboratory for analysis and are available in two sizes, 20 mL and 40 mL, and in clear and amber glass. 100 Screw caps and PTFE liners are included in the packs, which also contain 100 vials. Chromacol vials are available certified to three levels of cleanliness.

- *Class 100*. In this case the vials, 40-EPACSV, are initially cleaned, pre-assembled and packed under clean room conditions. This cleanliness level is suitable for most uses.
- *Class 200*. These vials, 40-EPACSV-PC, have an additional cleaning procedure so that the vials will be suitable for determination of trace amounts of environmental pollutants. These vials are accompanied by a certificate detailing the EPA Protocol employed during the cleaning process.
- *Class 300*. For the most stringent of analyses, where individual certification of each batch of vials is required. These vials, 40-EPACSV-PC3, are available by special order.

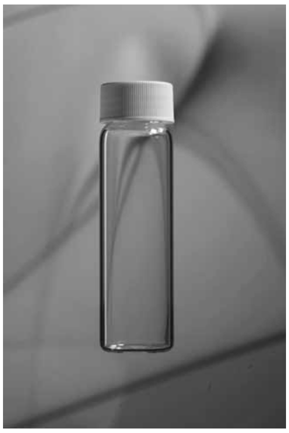

*Chromacol 40 mL EPA vial*

*For further information call Chromacol on 0845 7023964 or visit the Chromacol website at www.chromacol.com*

### Online analyser makes water sampling simple

Partech Instruments has introduced a purpose-designed online analyser for the automatic monitoring of potable, surface, sea, boiler and wastewater. The Micromac C/E is a microprocessor-controlled system that brings high levels of accuracy and efficiency to process monitoring and comes in Colorimetric (Micromac C) and Ion Selective Electrode (Micromac E) versions.

The consumption and replacement of reagents is recognized as being a major contribution to the operating costs, so in order to achieve economic operation the analyser will remain in stand-by mode between two measurements, without the consumption of any reagent. The measuring interval can be selected by the user to suit the application.

Partech has not overlooked the fact that samples can often be dirty and require the removal of contaminants prior to analysis. To this end, the options exist for sample dialysis and self-cleaning filtration. Using a single filtration unit, clean samples can be supplied for up to 10 separate analyses. Supplied with integral PLC, the self-cleaning filtration unit offers extended corrosion-free operation and comes ready for connection to the sample, waste and analyser sampling lines.

The range of features and options provided by the Micromac online analyser combine to provide end-users with an automated system that requires the minimum of operator interface once installed. Not only is calibration fully automatic, but also where off-scale samples are detected the analysis process is switched automatically to dilution mode.

Developed for industrial and environmental online applications, Micromac C/E is suitable for conditions where the robustness of the electronics, mechanics and hydraulic components is of paramount importance. Extended and reliable operation results from the complete separation of the electronics and hydraulics, together with the employment of a simple yet robust LFA (Loop Flow Analysis) system.

*Features and benefits*

- Fully automatic operation, including calibration
- Long autonomy, low maintenance, low running costs
- Low reagent consumption, short preparation time, low disposal costs
- Simple installation, with units factory built and application programmed
- Easy operation; no specialist training required
- Automatic dilution both for high range and off-scale samples
- Self-cleaning on-line filters package

*Applications*

In a multi-parameter configuration, the system may be used to analyse sequentially, $NH_3$ $NO_2$+ $NO_3$, $NO_2$ and $PO_4$. The inclusion of a UV digestion unit provides the capabilities to analyse sequentially Total P and Total N. Management of the sample digestion process is via integral software, which allows programming and control of each digestion step including volume, reagent(s) volume and UV digestion time.

For further information, contact: Angus Fosten, Partech Instruments, Charlestown, St Austell, Cornwall, PL25 3NN, UK; Tel: +44(0)1726 879800; E-mail: info@partech.co.uk; www.partech.co.uk; or Bryan Orchard, Clarendon Public Relations; Tel/Fax: 01295 760863; E-mail: orchard@primex.co.uk

